# Supplementary material for: Distributed genotyping and clustering of Neisseria strains reveal continual emergence of epidemic meningococcus over a century
Source: Nat Commun. 2023 Nov 24;14:7706. doi: 10.1038/s41467-023-43528-0 (PMC10673917; doi:10.1038/s41467-023-43528-0)
Supplement: Supplementary file 1 — Supplementary Information [file 41467_2023_43528_MOESM1_ESM.pdf]

Table of content:

Supplementary Figures **2**

Supplementary Figure 1 : HCCeval plots of Neisseria. 2

Supplementary Figure 2 : ANI groups of Nesseria genus 3

Supplementary Figure 3 : NJ tress of Neisseria 4

Supplementary Tables **5**

Supplementary Table 1: correspondence of species and HC1050&1130 5

Supplementary Table 2: inconsistency between HC760 groups and CCs 10

Supplementary Figures

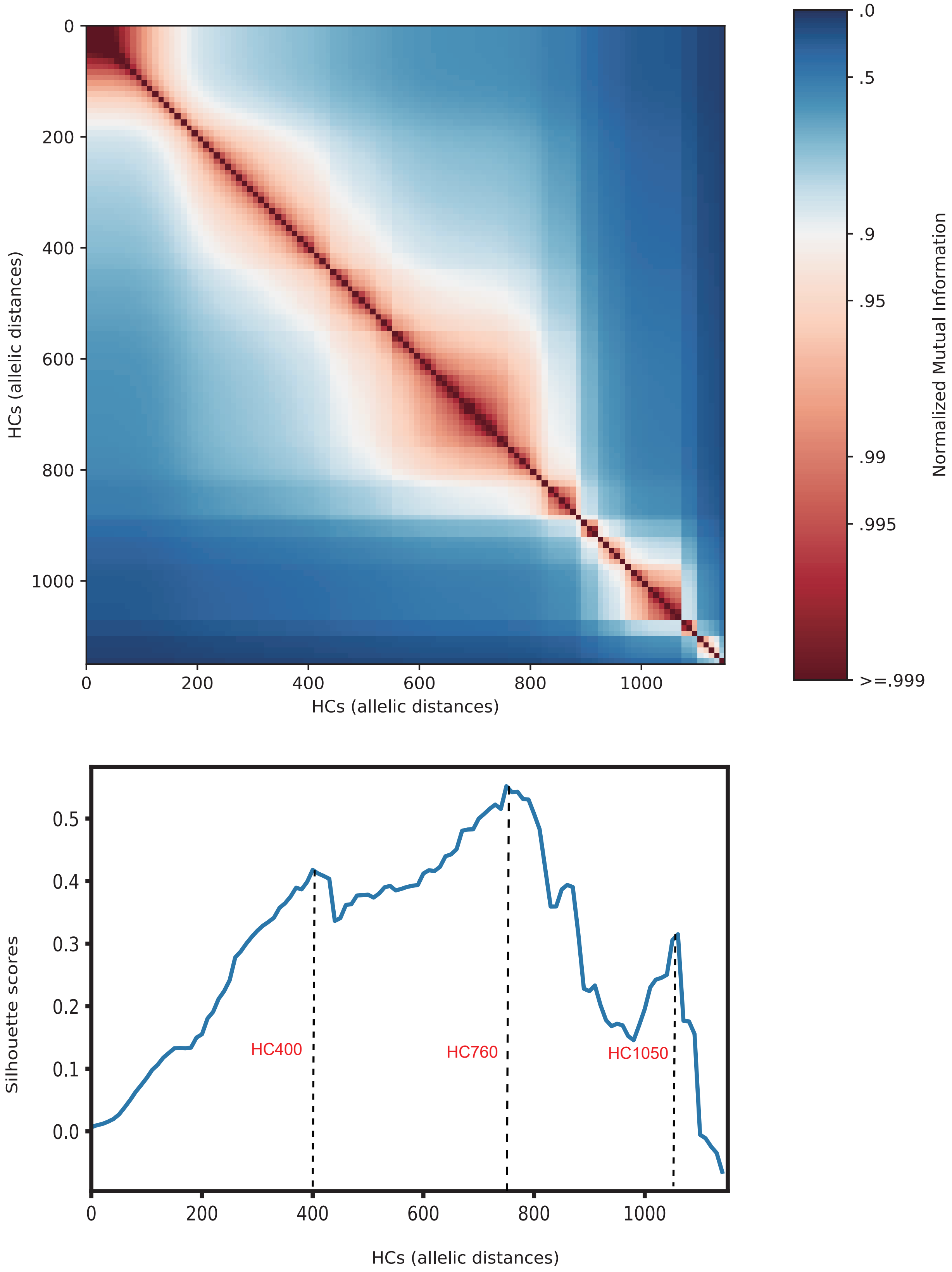

Supplementary Figure 1 : HCCeval plots of Neisseria. It evaluates the thousands of clustering levels generated by pHierCC and identifies potentially biologically meaningful clustering levels.

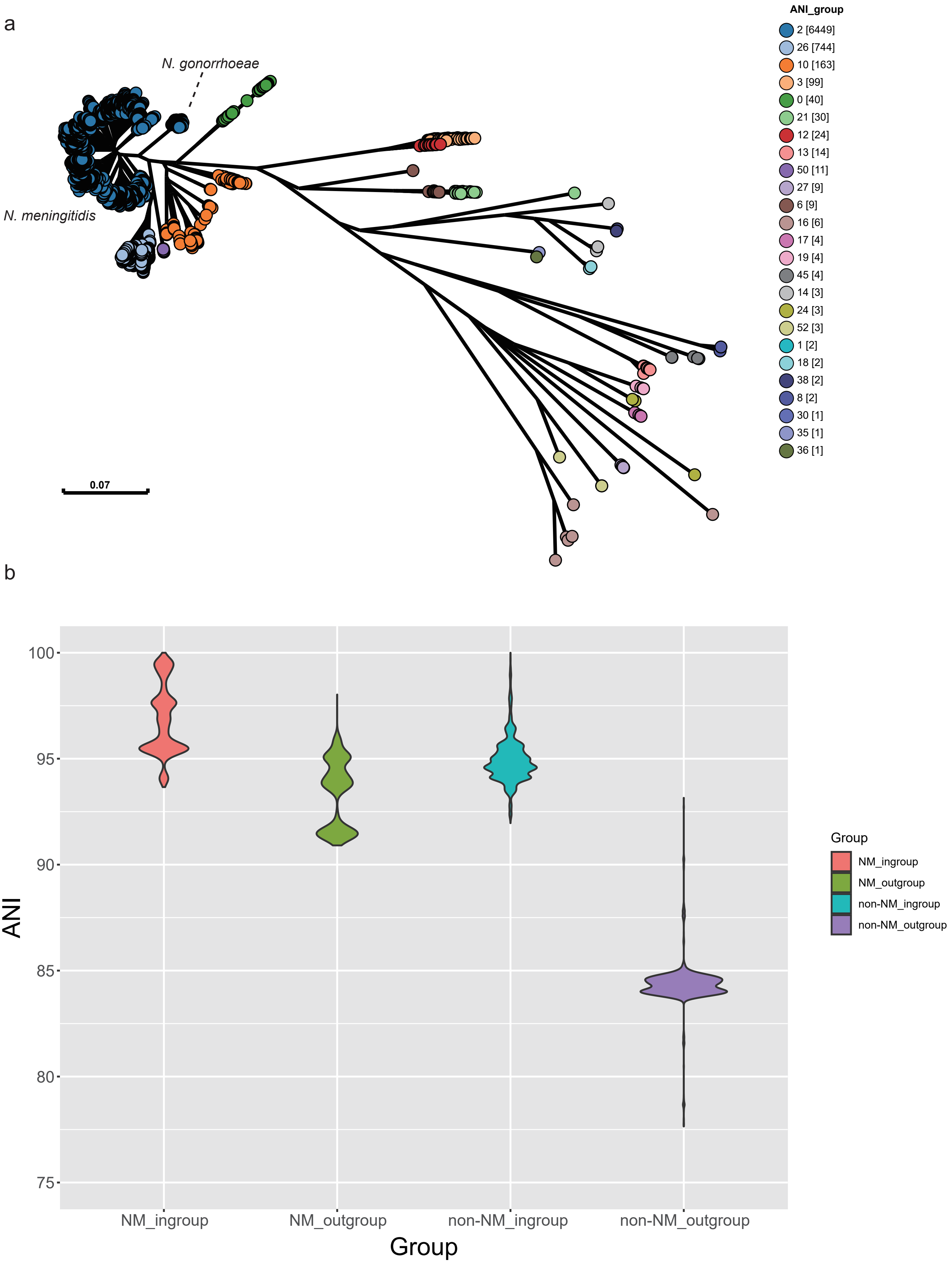

Supplementary Figure 2 : ANI groups of *Nesseria* genus (a) *Neisseria* species tree colored by 95% ANI groups which inaccurately grouped *N. meningitidis* and *N. gonorrhoeae* together. (b) Violin plot of ANI between four groups.

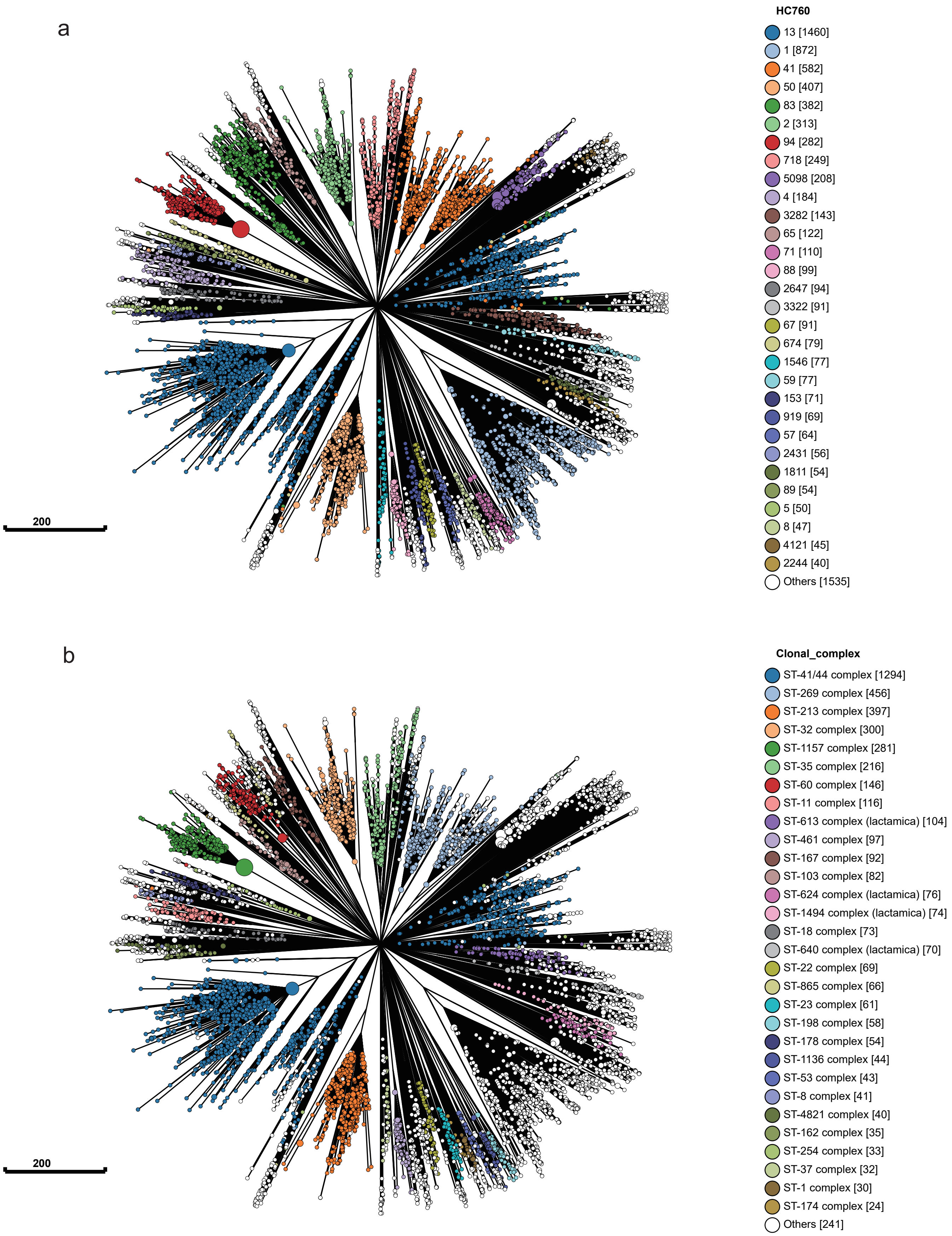

Supplementary Figure 3 : NJ tress of *Neisseria* colored by (a) HC760 groups and (b) Clonal_complex.

Supplementary Tables

Supplementary Table 1: correspondence of species and HC1050&1130

HC1050	HC1130	species	complex
1	1	*Neisseria gonorrhoeae*	NM
2	1	*Neisseria meningitidis*	NM
29	29	*Neisseria cinerea*	NM
32	1	*Neisseria lactamica*	NM
34	1	*Neisseria polysaccharea*	NM
55	1	*Neisseria polysaccharea*	NM
56	1	*Neisseria HC1050_56*	NM
960	1	*Neisseria cinerea*	NM
1706	1	*Neisseria bergeri*	NM
1717	1	*Neisseria blantyrii*	NM
1725	1	*Neisseria HC1050_1725*	NM
1731	1	*Neisseria viridiae*	NM
1817	29	*Neisseria cinerea*	NM
2356	29	*Neisseria cinerea*	NM
2409	1	*Neisseria polysaccharea*	NM
2596	1	*Neisseria polysaccharea*	NM
3129	1	*Neisseria cinerea*	NM
3474	29	*Neisseria cinerea*	NM
3540	1	*Neisseria lactamica*	NM
4178	1	*Neisseria cinerea*	NM
5474	1	*Neisseria polysaccharea*	NM
5786	1	*Neisseria polysaccharea*	NM
5808	1	*Neisseria polysaccharea*	NM
5817	29	*Neisseria cinerea*	NM
5936	1	*Neisseria HC1050_5936*	NM
6956	1	*Neisseria maigaei*	NM
6967	1	*Neisseria uirgultaei*	NM
7453	1	*Neisseria lactamica*	NM
11728	1	*Neisseria cinerea*	NM
17708	1	*Neisseria polysaccharea*	NM
20597	1	*Neisseria cinerea*	NM
20683	1	*Neisseria cinerea*	NM
25175	1	*Neisseria cinerea*	NM
25207	1	*Neisseria cinerea*	NM
25561	1	*Neisseria polysaccharea*	NM
25593	1	*Neisseria cinerea*	NM

26900	1	*Neisseria HC1050_26900*	NM
27067	1	*Neisseria HC1050_27067*	NM
37230	1	*Neisseria cinerea*	NM
37041	1	*Neisseria polysaccharea*	NM
27466	1	*Neisseria HC1050_27466*	NM
33927	1	*Neisseria cinerea*	NM
34662	1	*Neisseria cinerea*	NM
33936	1	*Neisseria polysaccharea*	NM
27742	29	*Neisseria cinerea*	NM
27812	1	*Neisseria cinerea*	NM
27837	27837	*Neisseria cinerea*	NM
27839	27839	*Neisseria cinerea*	NM
27927	27837	*Neisseria cinerea*	NM
35723	1	*Neisseria cinerea*	NM
39245	29	*Neisseria cinerea*	NM
39344	29	*Neisseria cinerea*	NM
39539	29	*Neisseria cinerea*	NM
39551	1	*Neisseria polysaccharea*	NM
9	9	*Neisseria HC1050_9*	non-NM
38946	38946	*Neisseria HC1130_38946*	non-NM
40198	40198	*Neisseria HC1130_40198*	non-NM
96	96	*Neisseria HC1130_96*	non-NM
180	180	*Neisseria animalis*	non-NM
39312	39312	*Neisseria animaloris*	non-NM
39345	39312	*Neisseria animaloris*	non-NM
42775	39312	*Neisseria animaloris*	non-NM
39409	39312	*Neisseria animaloris*	non-NM
38692	38692	*Neisseria arctica*	non-NM
58	58	*Neisseria bacilliformis*	non-NM
38756	58	*Neisseria bacilliformis*	non-NM
39310	39310	*Neisseria brasiliensis*	non-NM
39295	39295	*Neisseria canis*	non-NM
39289	39289	*Neisseria chenwenguii*	non-NM
39293	39293	*Neisseria dentiae*	non-NM
39031	39031	*Neisseria dumasiana*	non-NM
39348	39348	*Neisseria mucosa*	non-NM
39223	39031	*Neisseria dumasiana*	non-NM
39391	39031	*Neisseria dumasiana*	non-NM
39206	39031	*Neisseria dumasiana*	non-NM
5832	5832	*Neisseria elongata*	non-NM
39078	39078	*Neisseria elongata*	non-NM
39439	39439	*Neisseria elongata*	non-NM
38755	5832	*Neisseria elongata*	non-NM

41850	5832	*Neisseria elongata*	non-NM
39415	5832	*Neisseria elongata*	non-NM
38676	5832	*Neisseria elongata*	non-NM
41706	5832	*Neisseria elongata*	non-NM
39471	5832	*Neisseria elongata*	non-NM
39360	5832	*Neisseria elongata*	non-NM
39236	5832	*Neisseria elongata*	non-NM
38727	5832	*Neisseria elongata*	non-NM
39390	39390	*Neisseria iguanae*	non-NM
39214	39214	*Neisseria mucosa*	non-NM
39085	39085	*Neisseria mucosa*	non-NM
33	33	*Neisseria mucosa*	non-NM
39246	33	*Neisseria mucosa*	non-NM
39149	33	*Neisseria mucosa*	non-NM
75	33	*Neisseria mucosa*	non-NM
42835	33	*Neisseria mucosa*	non-NM
63	33	*Neisseria mucosa*	non-NM
53	53	*Neisseria mucosa*	non-NM
39229	28	*Neisseria mucosa*	non-NM
39600	28	*Neisseria mucosa*	non-NM
39163	28	*Neisseria mucosa*	non-NM
54	28	*Neisseria mucosa*	non-NM
38566	28	*Neisseria mucosa*	non-NM
39380	28	*Neisseria mucosa*	non-NM
28	28	*Neisseria mucosa*	non-NM
39191	28	*Neisseria mucosa*	non-NM
39057	28	*Neisseria mucosa*	non-NM
39008	28	*Neisseria mucosa*	non-NM
39151	28	*Neisseria mucosa*	non-NM
40135	40135	*Neisseria musculi*	non-NM
40179	62	*Neisseria shayeganii*	non-NM
62	62	*Neisseria shayeganii*	non-NM
1732	30	*Neisseria subflava*	non-NM
49956	49956	*Neisseria subflava*	non-NM
5840	159	*Neisseria subflava*	non-NM
50032	31	*Neisseria subflava*	non-NM
7677	31	*Neisseria subflava*	non-NM
19870	31	*Neisseria subflava*	non-NM
11451	31	*Neisseria subflava*	non-NM
51538	31	*Neisseria subflava*	non-NM
5901	159	*Neisseria subflava*	non-NM
48510	31	*Neisseria subflava*	non-NM
5971	31	*Neisseria subflava*	non-NM

48043	31	*Neisseria subflava*	non-NM
50105	31	*Neisseria subflava*	non-NM
946	159	*Neisseria subflava*	non-NM
5820	159	*Neisseria subflava*	non-NM
3448	31	*Neisseria subflava*	non-NM
31	31	*Neisseria subflava*	non-NM
8965	31	*Neisseria subflava*	non-NM
48115	48115	*Neisseria subflava*	non-NM
5880	30	*Neisseria subflava*	non-NM
27081	30	*Neisseria subflava*	non-NM
29796	30	*Neisseria subflava*	non-NM
27569	30	*Neisseria subflava*	non-NM
32887	30	*Neisseria subflava*	non-NM
34602	30	*Neisseria subflava*	non-NM
30	30	*Neisseria subflava*	non-NM
25330	31	*Neisseria subflava*	non-NM
25125	31	*Neisseria subflava*	non-NM
51428	31	*Neisseria subflava*	non-NM
159	159	*Neisseria subflava*	non-NM
42537	30	*Neisseria subflava*	non-NM
39003	39003	*Neisseria subflava*	non-NM
39358	31	*Neisseria subflava*	non-NM
39014	39014	*Neisseria subflava*	non-NM
40172	31	*Neisseria subflava*	non-NM
39056	31	*Neisseria subflava*	non-NM
3348	31	*Neisseria subflava*	non-NM
38898	31	*Neisseria subflava*	non-NM
39347	31	*Neisseria subflava*	non-NM
38976	31	*Neisseria subflava*	non-NM
40374	31	*Neisseria subflava*	non-NM
38909	31	*Neisseria subflava*	non-NM
39123	31	*Neisseria subflava*	non-NM
42721	42721	*Neisseria subflava*	non-NM
40311	31	*Neisseria subflava*	non-NM
39196	31	*Neisseria subflava*	non-NM
38457	159	*Neisseria subflava*	non-NM
38559	31	*Neisseria subflava*	non-NM
39095	159	*Neisseria subflava*	non-NM
5768	159	*Neisseria subflava*	non-NM
39089	31	*Neisseria subflava*	non-NM
39337	159	*Neisseria subflava*	non-NM
140	31	*Neisseria subflava*	non-NM
38811	31	*Neisseria subflava*	non-NM

39314	31	*Neisseria subflava*	non-NM
38787	31	*Neisseria subflava*	non-NM
39079	31	*Neisseria subflava*	non-NM
39117	31	*Neisseria subflava*	non-NM
37	31	*Neisseria subflava*	non-NM
38651	31	*Neisseria subflava*	non-NM
42598	159	*Neisseria subflava*	non-NM
64	64	*Neisseria wadsworthii*	non-NM
39317	64	*Neisseria wadsworthii*	non-NM
60	60	*Neisseria weaveri*	non-NM
39327	39327	*Neisseria weixii*	non-NM
40242	40242	*Neisseria zalophi*	non-NM
39208	39208	*Neisseria zoodegmatis*	non-NM
42849	39208	*Neisseria zoodegmatis*	non-NM

Supplementary Table 2: inconsistency between HC760 groups and CCs

a. One HC760 cluster correlates with two CCs

HC760	CC	No. genomes	Note
674	ST-254 complex	69	ST-1572C was derived from ST254C
	ST-1572 complex	44	
1546	ST-37 complex	59	ST-231C was derived from ST37C
	ST-231 complex	17	
2327	ST-282 complex	35	Both CCs formed a monophyletic group
	ST-212 complex	20	
71	ST-198 complex	1037	Both CCs formed a monophyletic group
	ST-1136 complex	132	
3	ST-5 complex	790	Both CCs formed a monophyletic group
	ST-4 complex	116	
2431	ST-364 complex	30	Both CCs formed a monophyletic group
	ST-334 complex	33	
4	ST-11 complex	6328	CCs intermixed in the SNP phylogeny
	ST-8 complex	283	

b. Two HC760 clusters correlates with one CC

HC760	CC	No. genomes	Note
35	ST-613 complex (lactamica)	213	Both HC760s formed a monophyletic group
3282		116	
1811	ST-624 complex (lactamica)	91	Both HC760s formed a monophyletic group
2244		27	
2647	ST-18 complex	100	Both HC760s formed a monophyletic group
7		17	
6623	ST-750 complex	12	CC was polyphyletic
919		8	

c. Real conflicts that can not be explained

HC760	CC	No. genomes	Note
65	ST-175 complex	601	
	ST-167 complex	481	
	ST-103 complex	8	ST103 C was polyphyletic
83		200	
32662		2	

<table>
<tr><td rowspan="3">83</td><td>ST-60 complex</td><td>469</td><td></td></tr>
<tr><td>ST-549 complex</td><td>7</td><td></td></tr>
<tr><td rowspan="2">ST-865 complex</td><td>133</td><td>ST865C was polyphyletic</td></tr>
<tr><td>2490</td><td>19</td><td></td></tr>
<tr><td rowspan="2">153</td><td>ST-162 complex</td><td>222</td><td></td></tr>
<tr><td rowspan="2">ST-2057 complex</td><td>32</td><td>ST2057C was polyphyletic</td></tr>
<tr><td>44461</td><td>2</td><td></td></tr>
</table>
