# Supplementary material for: Distributed genotyping and clustering of Neisseria strains reveal continual emergence of epidemic meningococcus over a century
Source: Nat Commun. 2023 Nov 24;14:7706. doi: 10.1038/s41467-023-43528-0 (PMC10673917; doi:10.1038/s41467-023-43528-0)
Supplement: Supplementary file 3 — Description of Additional Supplementary Files [file 41467_2023_43528_MOESM3_ESM.pdf]

Description of Additional Supplementary Files:

Supplementary Data 1: The metadata and genotypes for all 69,994 Neisseria strains in this study
