# Supplementary material for: Distributed genotyping and clustering of Neisseria strains reveal continual emergence of epidemic meningococcus over a century
Source: Nat Commun. 2023 Nov 24;14:7706. doi: 10.1038/s41467-023-43528-0 (PMC10673917; doi:10.1038/s41467-023-43528-0)
Supplement: Supplementary file 5 — Reporting Summary [file 41467_2023_43528_MOESM5_ESM.pdf]

nature portfolio

Corresponding author(s): Zhemin Zhou, Heng Li, Yahui Zhan

Last updated by author(s): Oct 3, 2023

Reporting Summary

Nature Portfolio wishes to improve the reproducibility of the work that we publish. This form provides structure for consistency and transparency in reporting. For further information on Nature Portfolio policies, see our Editorial Policies and the Editorial Policy Checklist.

Statistics

For all statistical analyses, confirm that the following items are present in the figure legend, table legend, main text, or Methods section.

n/a	Confirmed	
[ ]	[x]	The exact sample size (*n*) for each experimental group/condition, given as a discrete number and unit of measurement
[x]	[ ]	A statement on whether measurements were taken from distinct samples or whether the same sample was measured repeatedly
[x]	[ ]	The statistical test(s) used AND whether they are one- or two-sided<br>*Only common tests should be described solely by name; describe more complex techniques in the Methods section.*
[x]	[ ]	A description of all covariates tested
[ ]	[x]	A description of any assumptions or corrections, such as tests of normality and adjustment for multiple comparisons
[ ]	[x]	A full description of the statistical parameters including central tendency (e.g. means) or other basic estimates (e.g. regression coefficient) AND variation (e.g. standard deviation) or associated estimates of uncertainty (e.g. confidence intervals)
[x]	[ ]	For null hypothesis testing, the test statistic (e.g. *F*, *t*, *r*) with confidence intervals, effect sizes, degrees of freedom and *P* value noted<br>*Give P values as exact values whenever suitable.*
[ ]	[x]	For Bayesian analysis, information on the choice of priors and Markov chain Monte Carlo settings
[ ]	[x]	For hierarchical and complex designs, identification of the appropriate level for tests and full reporting of outcomes
[x]	[ ]	Estimates of effect sizes (e.g. Cohen's *d*, Pearson's *r*), indicating how they were calculated

Our web collection on statistics for biologists contains articles on many of the points above.

Software and code

Policy information about availability of computer code

Data collection	Using SRA Toolkit (2.8.0) to download genomes and short reads of Neisseria from NCBI
Data analysis	The folowing software were used in the analysis: EToKi v1.2, Kssd v1.1, fetchMGs v1.3, prokka 1.14.5, PEPPAN v1.0.5, FastANI v1.32,GrapeTree v1.5.0,pHierCC v1.3.2, BactDating v1.1, TreeTime v0.9, cgMLSA v1.0, ERaBLE 1.0, ASTRAL-Ill 5.7.1, RecHMM 08d7382, ASTRID 3, DTy v1.0, R v4.1.4, R packages (ggplot23.3.3, dplyrl.0.6, skygrowth c375aac)

For manuscripts utilizing custom algorithms or software that are central to the research but not yet described in published literature, software must be made available to editors and reviewers. We strongly encourage code deposition in a community repository (e.g. GitHub). See the Nature Portfolio guidelines for submitting code & software for further information.

Data

Policy information about availability of data

All manuscripts must include a data availability statement. This statement should provide the following information, where applicable:

- Accession codes, unique identifiers, or web links for publicly available datasets
- A description of any restrictions on data availability
- For clinical datasets or third party data, please ensure that the statement adheres to our policy

Source data are provided with this paper. The raw reads of 149 strains from this study have been deposited in the GSA under accession code PRJCA015852 [https://ngdc.cncb.ac.cn/bioproject/browse/PRJCA015852]. The assembled genome sequences have been deposited in theThe assembled genome sequences have been deposited in the Genebank database under accession code PRJNA1022841 [https://www.ncbi.nlm.nih.gov/bioproject/PRJNA1022841], and are also available in

figshare at https://tinyurl.com/assembledNeisseria. The species tree of Neisseria is available at https://tinyurl.com/Neisseria-species. All accessions of genomes from the public database are included in Supplementary Data 1. The metadata supporting the conclusions of this article are also included in Supplementary Data 1. Source data for the figures (including supplementary figures) are provided in this paper. The reference sequences of core genes in the dcgMLST scheme, as well as the nomenclature results and HierCC profiles of all genomes, are provided as part of the DTy pipeline at https://github.com/ADSGF203com/DTy/tree/master/db/Neisseria

Research involving human participants, their data, or biological material

Policy information about studies with human participants or human data. See also policy information about sex, gender (identity/presentation), and sexual orientation and race, ethnicity and racism.

Reporting on sex and gender	The sex and gender were not considered in study design so most of this information has not been collected except 2 strains from a mother and a daughter, and 4 strains from a female patient and her mother, grandfather and grandmother.
Reporting on race, ethnicity, or other socially relevant groupings	All participants are Chinese.
Population characteristics	Not applicable
Recruitment	This study included data from the Suzhou Center for Disease Control and Prevention (CDC) in Suzhou, China. Patientinformation was collected if they tested positive for Neisseria meningitidis and biochemical testing. This was done for alcases. All tests were conducted as advised by the treating physician, and the data were colected as a part of passivesurveillance. There is no possibility of self selection bias.
Ethics oversight	This study was approved by the Institutional Review Board (IRB) of the Jiangsu Provincial Center for Disease Control and Prevention. The “Guidance of the Ministry of Science and Technology (MOST) for the Review and Approval of Human Genetic Resources” was not required for the study on human participants in accordance with the local legislation and institutional requirements. Written informed consent to participants in this study was provided by adult participants or the participants' legal guardians of child participants. No compensation was provided to the participants.

Note that full information on the approval of the study protocol must also be provided in the manuscript.

Field-specific reporting

Please select the one below that is the best fit for your research. If you are not sure, read the appropriate sections before making your selection.

[x] Life sciences [ ] Behavioural & social sciences [ ] Ecological, evolutionary & environmental sciences

For a reference copy of the document with all sections, see nature.com/documents/nr-reporting-summary-flat.pdf

Life sciences study design

All studies must disclose on these points even when the disclosure is negative.

Sample size	We included all 69,994 publicly available Neisseria genomes, and 149 genomes from all available N. meningitidis isolates in Suzhou, China in 1975-2021, resulting in the most comprehensive collection so far for the investigation of historic epidemics.
Data exclusions	Genomic assemblies with N50 values < 0.2 MB or > 15 MB, and total sizes < 1.9 or > 4 MB were retained
Replication	Bioinformatic analyses were described in sufficient detail to reproduce the findings with the publicly available sequence data. We have given out all the source data as described in the manuscript. And the scripts for dcgMLST has been released as the DTy pipeline, which is also included in the manuscript.
Randomization	Randomization was not required for the isolate selection process since (1) our goal was to collect all available Neisseria genomes to construct a dataset that represents the population to date, and (2) isolates were not allocated to different experimental groups.
Blinding	During the data acquisition and analysis, we worked with the isolate identifiers or accession codes to remove any potential for bias that might have arisen from knowledge of the specific samples being analyzed.

Reporting for specific materials, systems and methods

We require information from authors about some types of materials, experimental systems and methods used in many studies. Here, indicate whether each material, system or method listed is relevant to your study. If you are not sure if a list item applies to your research, read the appropriate section before selecting a response.

Materials & experimental systems

n/a	Involved in the study
☒	☐ Antibodies
☒	☐ Eukaryotic cell lines
☒	☐ Palaeontology and archaeology
☒	☐ Animals and other organisms
☒	☐ Clinical data
☒	☐ Dual use research of concern
☒	☐ Plants

Methods

n/a	Involved in the study
☒	☐ ChIP-seq
☒	☐ Flow cytometry
☒	☐ MRI-based neuroimaging
